# Three-Phase Three-Dimensional Electrochemical Process for Efficient Treatment of Greywater

**DOI:** 10.3390/membranes12050514

**Published:** 2022-05-12

**Authors:** Weiyang Li, Wei Wang, Peng Zhang

**Affiliations:** 1 State Key Laboratory of Separation Membrane and Membrane Process, School of Material Science and Engineering, Tiangong University, Tianjin 300387, China; 1910210504@tiangong.edu.cn; 2 Jiangsu Longmem Environmental Technology Co., Ltd., Changzhou 213000, China; 15234838139@163.com

**Keywords:** three-dimensional electrode, COD_Cr_, greywater, response surface methodology

## Abstract

Water shortages around the world have intensified the search for substitute sources. Greywater can serve as a solution for water requirements. Compared to two-dimensional electrochemical processes for water treatment, the addition of particle activated carbon enhances the conductivity and mass transfer or the adsorption of pollutants in a three-dimensional (3D) electrochemical process. The large specific surface areas of these particles can provide more reactive sites, resulting in a higher removal efficiency. In this study, the treatment of greywater by the electro-Fenton (E-Fenton) method was carried out in a 3D electrolytic reactor. The effects of the operating conditions, such as electrode spacing, applied voltage, treatment time, and activated carbon loading, on the efficacy of the E-Fenton process were investigated, and the corresponding optimum conditions were found to be 7 cm, 9 V, 2 h, and 10 g. The results showed that COD_Cr_ removal of greywater treated using the 3D electrochemical process was 85%. With the help of the Box–Behnken experiment design and the response surface methodology, the parameters were optimized to determine the optimal conditions. The results of the response surface analysis were consistent with the experimental results. The above findings illustrate that the proposed three-phase 3D electrochemical process is feasible for the efficient treatment of greywater.

## 1. Introduction

With the ongoing global progress in social and economic development, the problem of water shortages is becoming increasingly alarming, especially due to the unwise and inefficient use of water resources [1,2,3]. The World Water Council projects that global water consumption will increase by approximately 50% by 2034 [4]. In China, greywater accounts for approximately 30% of urban domestic wastewater [5]. Since it is moderately polluted, it can be recycled and reused. From an environmental perspective, it is wiser to recycle greywater than further pollute urban wastewaters [6]. Draining greywater directly (i.e., without treatment) into a drainage system will cause pollution of natural water system [7,8,9]. Moreover, it will produce destructive and cumulative biological diseases and have a greater impact on human health [10,11]. For example, most cases of enteric virus infections originate from contaminated drinking water resources, recreational waters, and foods contaminated by sewage and sewage effluent waters [12,13].

Within this context, conventional biological treatment does not always achieve satisfactory results, and traditional physicochemical methods are relatively expensive, ineffective, or may lead to secondary contamination. For example, the dissolved air flotation method [14,15,16] involves injection of a large number of dense bubbles into treated wastewater, whereupon impurities adhere to the bubbles, effectively forming a liquid with a density less than that of water. The primary disadvantage of this treatment method is that it is difficult to directly contact the suspended sludge, which results in secondary sludge formation. In coagulation–flocculation treatment methods [17,18], colloidal particles in contaminated water collide and agglomerate, thus forming larger particles or flocs. However, these methods are expensive and ineffective at removing anionic detergents and pathogenic pollutants from greywater [19].

Advancements in water treatment technologies enable efficient treatment of wastewater [20,21,22]. Electrochemical technologies are a huge improvement in the field of wastewater treatment because of their high efficiency, environmental protection, and versatility. Despite these advantages, conventional two-dimensional (2D) electrochemical electrodes have a mass transfer limitation, small space–time yield, and low area–volume ratio. The development of three-dimensional (3D) electrochemical electrodes provides an outstanding solution to the above shortcomings that limit the application of 2D electrodes. Compared with conventional electrochemical technologies, 3D electrochemical processes can overcome the shortcomings of plane electrode design due to the increased electrode surface area per reactor unit volume and higher throughput. This enables high current efficiency, improved productivity, compact design, decolorization, and efficient removal of heavy metals. Moreover, the biochemical characteristics of processed wastewater can also be improved. High treatment capacity, lack of secondary pollution, and mild reaction conditions are among the other advantages of this technology [23,24].

Table 1 presents a comparison of the performances of a 3D electrochemical process and other physiochemical treatment processes for different target pollutants. It clearly illustrates the high efficiency of the 3D electrochemical process in COD_Cr_ removal. However, the particle electrode may lose its adsorption capacity and catalytic activity due to the accumulation of pollutants on particle surfaces over continuous runs [25]. In general, 3D electrochemical technology stimulates the further development of electrocatalysis technology with the aim of applying it to treatment of highly concentrated wastewaters [26,27,28]. This can also help solve the problem of the treatment and reuse of greywater [29,30,31].

Herein, the experiments were carried out in a homemade 3D electrode reactor. The effects of the interelectrode spacing, voltage, treatment time, and activated carbon loading on the performance of greywater treatment were investigated. The feasibility and efficiency of the 3D electrochemical process for the treatment of greywater were also verified. Additional analysis aimed at the optimization of the process parameters was performed using the response surface method and the Box–Behnken design [37,38,39]. These findings are expected to encourage the application of 3D electrochemical technology in greywater treatment. A three-dimensional process can serve as a pretreatment process to increase the biodegradability of effluent. This will be a trend in future development.

## 2. Materials and Methods

### 2.1. Materials

Cholesterol (C_27_H_46_O, BR), fatty acid (C_n_H_2n_O_2_, AR), calcium chloride (CaCl_2_, AR), potassium dihydrogen phosphate (KH_2_PO_4_, AR), lactic acid (C_3_H_6_O_3_, AR), and glucose (C_6_H_12_O_6_, AR) were purchased from Tianjin Guangfu Fine Chemical Research Institute (China). Sodium chloride (NaCl, AR) was purchased from Tianjin Wind Ship Chemical Reagent Technology Co., Ltd. (Tianjin, China). Magnesium sulfate (Mg_2_SO_4_, AR) was purchased from Tianjin Standard Technology Co., Ltd. (Tianjin, China). Potassium chloride (KCl, AR) was purchased from Tianjin Yingda Rare and Precious Chemical Reagent Factory (Tianjin, China). Urea (CH_4_N_2_O, AR) was purchased from Tianjin Shentai Chemical Reagent Co., Ltd. (Tianjin, China). The shower gel and activated carbon were purchased from a local market. Ultrapure water was produced in laboratory.

### 2.2. Preparation of Simulated Greywater

According to a certain proportion (see Table 2), the reagents were weighed and dissolved in pure water and then mixed well under ultrasound. The simulated greywater was characterized by a high concentration and complex composition. The characteristics of the simulated greywater water are listed in Table 3, which was provided by Jiangsu Longmem Environmental Technology Co., Ltd. (Changzhou, China).

### 2.3. The Electrolysis System

The mechanism of the electro-Fenton (E-Fenton) method [40] involves the reduction of O_2_ to H_2_O_2_ at the cathode, which produces •OH radicals via the subsequent Fenton reaction involving Fe^2+^. These radicals then oxidize organic matter to CO_2_ and H_2_O or small organic molecules [41,42].

(1) $$O_{2}+2e^{-}+2H^{+}\;\to \;H_{2}O_{2}$$

(2) $$H_{2}O_{2}+Fe^{2+}\;\to \;Fe^{3+}+OH^{-}+•OH^{-}$$

The dioxygen required for Reaction (1) can be supplied to the cathode of the electrolysis reactor by means of external aeration or produced on the anode according to Reactions (3) or (4).

(3) $$2H_{2}O\;\to \;O_{2}+4H^{+}+4e^{-}$$

(4) $$4OH^{-}\;\to \;O_{2}{}^{}+2H_{2}O+4e^{-}$$

**Figure 1:**
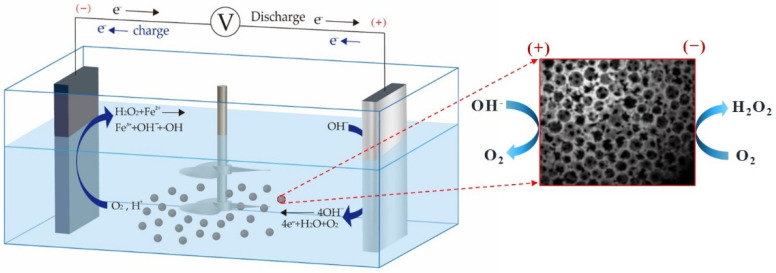
Schematic illustration of the electrocatalytic reactor.

The constructed E-Fenton system with 3D electrodes is capable of degrading pollutants in different ways [43,44]. In addition to the direct oxidation at the anode, the cathode has strong adsorption and catalytic properties, which can reduce the dissolved oxygen present in the system to H_2_O_2_. In the presence of H_2_O_2_ and added Fe^2+^ ions, •OH radicals are generated during the Fenton reaction and oxidize the organic matter. In addition, the electric field between the main electrodes can also cause the activated carbon particles to be charged with positive and negative charges due to the fact of electrostatic induction, forming an independent miniature electrolytic cell. As a result, electrochemical redox reactions can proceed simultaneously on the surface of each particle. The mechanism of the electrolysis reaction is presented in Figure 1.

### 2.4. Electro-Fenton Process for Greywater Using Three-Dimensional Electrodes

#### 2.4.1. Pretreatment of the Particle Electrodes

In this experiment, the activated carbon particles were repeatedly washed several times beforehand in order to avoid the adsorption effect of the activated carbon on the effectiveness of the 3D electrodes in treating greywater. The cleaned activated carbon was ultrasonically treated in the greywater. Each ultrasonic treatment step was carried out for 3 h. After three repeated ultrasonic treatments, the adsorption of activated carbon was considered to have reached saturation.

#### 2.4.2. Three-Dimensional Electrodes

In this experiment, a homemade 3D electrode reactor was used. The reactor was built from transparent organic glass and had a usable volume of 1.5 L. A stainless-steel plate was used as the anode, and a graphite plate with a thickness of 2 mm and an effective treatment area of 70 cm^2^ was used as the cathode.

The prepared greywater was added to the catalytic reactor, followed by the addition of the weighed quantity of granulated activated carbon. The experimental device is shown in Figure 1.

### 2.5. Electro-Fenton Process for Greywater Using Three-Dimensional Electrodes

The analysis methods of water quality correlation are shown in Table 3. The COD_Cr_ of the greywater was determined by the potassium dichromate method [45] using a COD_Cr_ detector (HACH DR3900, Loveland, CO, USA). The conductivity of the greywater was analyzed by a conductivity meter (HACH HQ40d, Loveland, CO, USA). The voltage in the 3D electrode system was provided by a DC regulated power supply (GWINSTEK GPS-3030DD).

## 3. Results and Discussion

### 3.1. The Effect of Electrode Spacing on the Degree of Greywater Treatment

**Figure 2:**
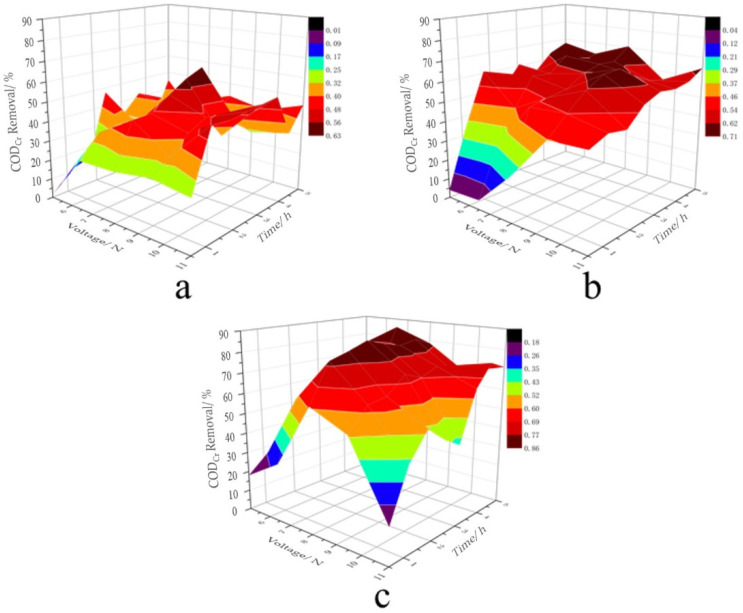
Effect of the process parameters on COD_Cr_ at different values for the interelectrode spacing: (**a**) 3; (**b**) 5; (**c**) 7 cm.

The effectiveness of the proposed treatment in decreasing chemical oxygen demand (COD_Cr_) and other characteristics of greywater was studied by varying the process parameters within reasonable limits: voltage, 5–11 V; treatment time, 0–5 h; interelectrode spacing of 3, 5, and 7 cm; activated carbon loading of 10 g. The results are presented in Figure 2.

As can be seen in Figure 2, with the increase in interelectrode spacing, the COD_Cr_ removal of greywater increased [46]. When the other variables were kept constant, this was mainly due to the small distance between electrodes and the low energy of the electrolytic system, which affected the mass transfer efficiency. With the increase in the distance between electrodes, the mass transfer process became more intensive due to the concentration gradient between organic matter and solution. This improved the efficiency of the degradation of the organic pollutants.

### 3.2. Effect of Different Factors on the Degree of Greywater Treatment

#### 3.2.1. The Treatment Time

**Figure 3:**
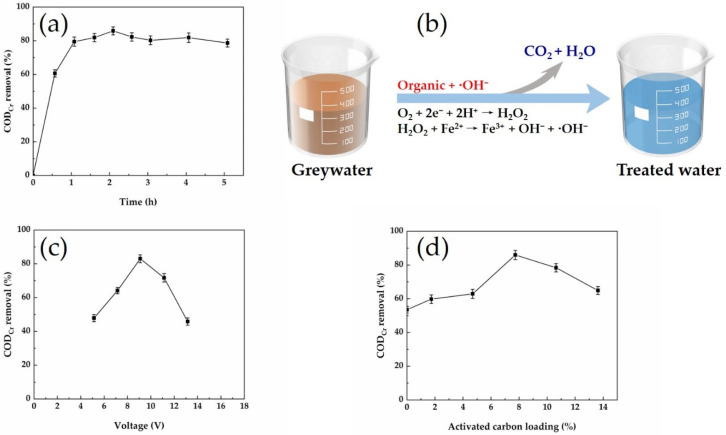
The effects of the process parameters on COD_Cr_ removal: (**a**) treatment time; (**b**) the COD_Cr_ degradation process; (**c**) voltage; (**d**) activated carbon loading.

At early stages of the treatment process, COD_Cr_ removal increased rapidly with the increase in processing time. After a period of time, the COD_Cr_ removal basically remained unchanged. This was mainly because the concentration of organic matter in the system gradually decreased during electrolysis and the catalytic effect diminished. The results are shown in Figure 3a.

#### 3.2.2. Applied Voltage

With the increase in voltage, the COD_Cr_ removal efficiency initially tended to increase but then decreased. The voltage affected the amount and rate of •OH production. If the voltage was too small, the voltage on the particle electrode was insufficient, resulting in less •OH and a weaker catalytic effect. Thus, the voltage at the particle electrode could not reach the anode or cathode. Contact between the particle electrode causes short circuiting, which reduces the efficiency of the electrolytic process, and a high voltage. The electrodes were subject to side reactions, such as hydrogen evolution reactions, which affected the current efficiency and reduced the effectiveness of the COD_Cr_ removal.

#### 3.2.3. Activated Carbon Loading

With the increase in activated carbon loading, the COD_Cr_ removal from the greywater by the 3D electrodes showed a trend of first increasing and then decreasing but basically remained above 60%. The highest COD_Cr_ removal of 85.7% was achieved at 10 g of activated carbon. This was mainly because the amount of particle electrodes added affects the electrolysis efficiency of the system. The lower the activated carbon loading, the fewer reaction sites are involved in the reaction, resulting in a lower electrolysis efficiency of the system. When the activated carbon loading increased, there were more reaction sites in the system, shortening the mass transfer distance between pollutants. However, when the activated carbon loading was excessive, the increased resistance caused the system to have side effects, resulting in a higher temperature of the system, thus reducing the electrolysis rate.

From Table 4, it can be seen that the 3D electrochemical process had a higher COD_Cr_ removal efficiency than the conventional 2D electrochemical process in the treatment of wastewater.

### 3.3. Changes in the Electrical Conductivity of Greywater during Treatment

**Figure 4:**
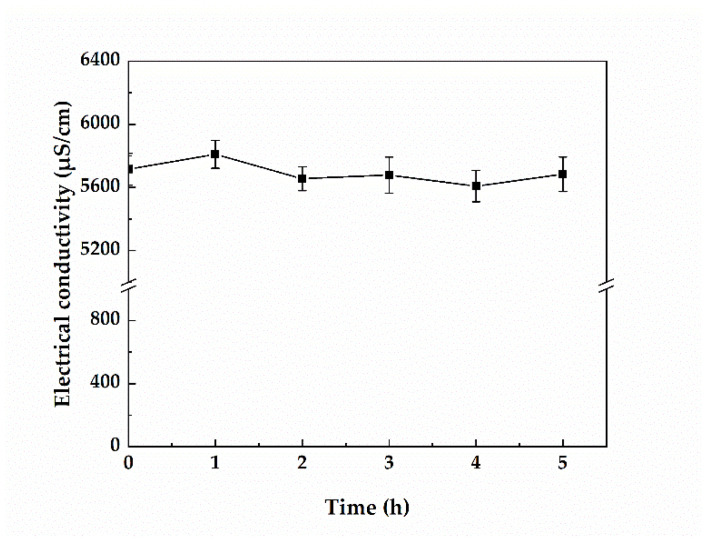
Effect of treatment time on the electrical conductivity (the activated carbon loading was 10 g, the interelectrode spacing was 7 cm, and the voltage was 9 V).

The conductivity of greywater changes depending on its salt content. As the electrocatalytic process proceeded, more of the solute in solution was ionized, and the conductivity increased. However, in general, the change in conductivity is small and negligible (Figure 4).

### 3.4. Changes in the Turbidity of Greywater during Treatment

**Figure 5:**
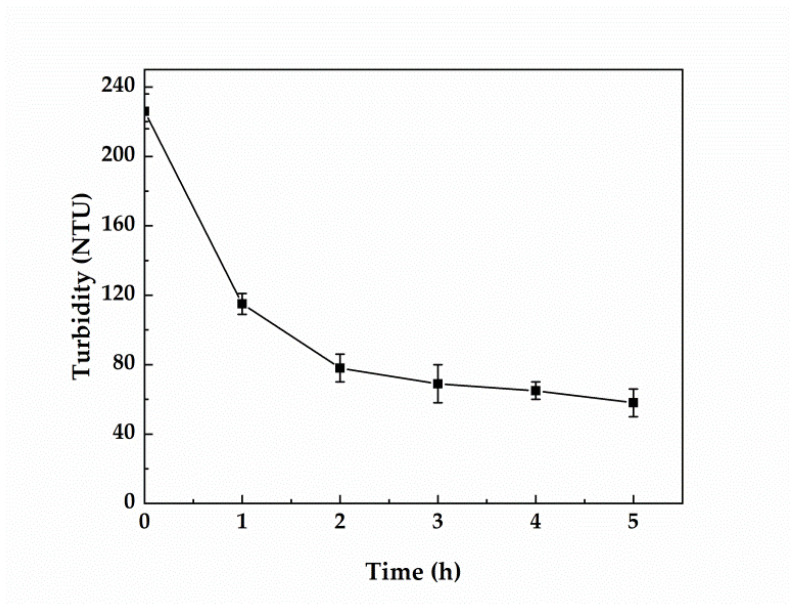
Effect of treatment time on the turbidity of greywater (the activated carbon loading was 10 g, the interelectrode spacing was 7 cm, and the voltage was 9 V).

As can be seen in Figure 5, the turbidity of the treated water decreased rapidly during the first 1–2 h of treatment. However, it started to decrease more gradually during the subsequent 3 h of treatment (i.e., 2–5 h since the beginning of the process). The greywater turbidity was significantly reduced during the electrocatalytic process due to the loose and porous structure of activated carbon [51] (Figure 1). The atomic force field on its surface was not saturated with surface energy and, thus, the surface energy was reduced via adsorption of molecules. As the treatment time increased, the turbidity of treated water decreased, and the adsorption capacity of activated carbon gradually reached saturation.

### 3.5. Box–Behnken Design and Response Surface Methodology

Through previous experiments, it is found that when the interelectrode spacing was 7 cm, the voltage was 9 V, the activated carbon loading was 10 g, and the process period was 2 h, the COD_Cr_ of greywater treated using the 3D electrode decreased by 85%. Further optimization of the process parameters—voltage, treatment time, and activated carbon loading—was performed using the Box–Behnken experimental design and the response surface methodology (RSM). The values for these three factors in run 3, as obtained from the steepest ascent path (Table 5), were taken as the central points. The respective low and high levels for each factor were coded as shown in Table 6. Fitting the experimental data using regression analysis gave the following second-order polynomial equation:

(5) $$Y=85.68-5.42A+8.81B+2.41C+0.57AB+0.53AC+2.05BC-15.82A^{2}-8.94B^{2}-11.64C^{2}$$

where *Y* is the predicted COD_Cr_ removal; *A*, *B*, and *C* are the code variables for voltage, time, and activated carbon loading, respectively.

The obtained F-value of 1671.80 implies that the model was significant. There was only a 0.01% chance that such a large F-value was due to the fact of noise. Based on the F-values for *A*, *B*, and *C*, the relative influence of the three factors on COD_Cr_ removal followed the order: time > voltage > activated carbon loading. The “predicted R-squared” value of 0.9938 was in reasonable agreement with the “adjusted R-squared” value of 0.9989”, i.e., the difference was less than 0.2. The *p*-value is usually used to test the significance of a variable. The smaller the *p*-value, the more significant the corresponding variable. As shown in Table 7, the *p*-values for *A*, *B*, and *C* were much less than 0.0001, indicating that the voltage, time, and activated carbon loading are important process parameters influencing the removal of COD_Cr_.

### 3.6. Results of the Response Surface Optimization of the Proposed Greywater Treatment Method

**Figure 6:**
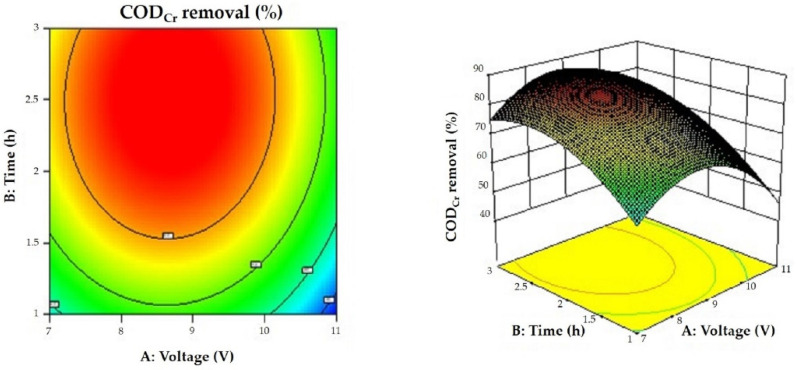
Response surface plot and the corresponding contour plot showing the effects of voltage and time on COD_Cr_ removal. The level of activated carbon loading was 10.28 g.

**Figure 7:**
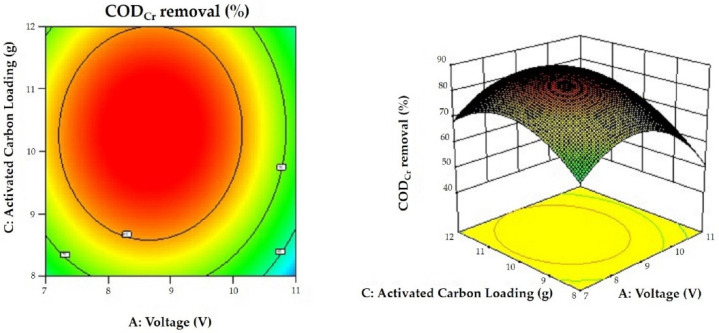
Response surface plot and the corresponding contour plot showing the effects of voltage and activated carbon loading on COD_Cr_ removal. The time level was 2.50 h.

**Figure 8:**
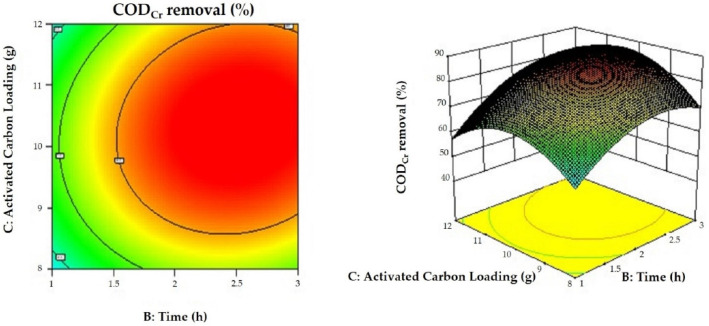
Response surface plot and the corresponding contour plot showing the effects of time and activated carbon loading on COD_Cr_ removal. The voltage was 8.68 V.

The response surfaces are presented in Figure 6, Figure 7 and Figure 8 in the form of 3D surfaces and contour plots. As can be seen from the figures, the response surfaces were convex with each plot representing an optimal condition, and the variables had maxima. In addition, Figure 8 shows a better ellipse, indicating better interaction between the variables representing time and activated carbon loading. However, the interaction between voltage, time, and activated carbon loading was not significant, which is consistent with the results of the response surface analysis. The response surface analysis showed that the greywater COD_Cr_ decreased by 88.51% at a voltage of 8.68 V, treatment duration of 2.50 h, and an activated carbon loading of 10.28 g.

## 4. Conclusions

A method for greywater treatment using 3D electrodes was developed and applied with good results. Single-factor experiments show that the treatment duration, voltage, and activated carbon loading are three key factors influencing the COD_Cr_ level of greywater. The Box–Behnken design and the response surface method were used for more advanced optimization of the three factors listed above and to determine the optimal reaction conditions. Specifically, it was found that for a voltage of 8.7 V, a treatment duration of 2.5 h, and an activated carbon loading of 10.3 g, the COD_Cr_ decreased by 88.5%. When the interelectrode spacing, voltage, treatment duration, and activated carbon loading were 7 cm, 9 V, 2 h, and 10 g, respectively, the COD_Cr_ of treated greywater decreased by 85.6%. The experimental values and the predicted values coincided well.

## Figures and Tables

**Table 1:** Comparison of COD_Cr_ ^1^ removal efficiency of different target pollutants by different physiochemical treatment processes.

Treatment Process	Target Pollutants	Key Processing Conditions	COD_Cr_ Removal (%)	Reference
C–ISF ^2^	Greywater	CS ^9^ = 2.97 mm, Gravel = 8.38 mm	80	[32]
PE-MBR ^3^	Textile wastewater	MR ^10^ = 462 cm^2^	52.0	[33]
ELA-MBR ^4^	Pharmaceutical wastewater	MR = 40 cm^2^	50	[34]
DEC ^5^	Industrial wastewater	CC ^11^ = 1000 mg·L^−1^, *j* ^12^ = 10 mA·cm^−2^, pH = 6	79.1	[35]
2-DET ^6^	PSM ^8^ wastewater	*j* = 30 mA·cm^−2^, HRT ^13^ = 60 min, pH = 8	57.2	[36]
3-DET ^7^	Paper mill wastewater	*j* = 167 mA·cm^−2^, pH = 11, *T* = 20 °C	86.3	[27]

^1^ Chemical Oxygen Demand. ^2^ Coagulation and intermittent sand filtration. ^3^ Photocatalytic electrolysis membrane reactor. ^4^ External loop airlift membrane bioreactor. ^5^ Divided electrolysis cell. ^6^ Two-dimensional electrochemical technology. ^7^ Three-dimensional electrochemical technology. ^8^ Fish sauce manufacturing. ^9^ Coarse sand. ^10^ Membrane area. ^11^ Chloride concentration. ^12^ Current density. ^13^ Reaction time.

**Table 2:** Composition of the simulated greywater.

Components	Concentration (g/L)	Components	Concentration (g/L)
Glucose	1.8	Lactic acid	0.7
Urea	1.7	KH_2_PO_4_	0.4
NaCl	2.1	Fatty acids	8.0
KCl	0.8	Mg_2_SO_4_	0.2
CaCl_2_	0.1	Shower gel	1.0
Cholesterol	0.5	-	-

**Table 3:** Characteristics of the simulated greywater.

pH	Turbidity (NTU ^1^)	COD_Cr_ ^2^ (mg/L)	TDS ^3^ (μS/cm)
3–3.5	189–227	420–995	3904–6389

^1^ Nephelometric turbidity unit. ^2^ Chemical oxygen demand. ^3^ Total inorganic carbon.

**Table 4:** Comparison of COD_Cr_ removal efficiency between 2D and 3D electrode reactors.

Reactor Types	Wastewaters	Conditions	COD_Cr_ Removal (%)	Reference
2D ^1^	HOR ^3^ wastewater	*j* ^4^ = 30 mA·cm^−2^, *T* = 60 °C, HRT ^5^ = 100 min	30.8	[47]
2D	Indigo wastewater	*U* = 9 V, HRT = 60 min, NC ^6^ = 5 g/L	60.3	[48]
2D	Dairy wastewater	*j* = 2730 mA·cm^−2^, pH = 7, HRT = 50 min	70	[49]
2D	Textile wastewater	*j* = 15 mA·cm^−2^, pH = 5, HRT = 120 min	77.7	[50]
3D ^2^	Greywater	*U* = 9 V, GAC ^7^ = 10 g, ES ^8^ = 7 cm, HRT = 120 min	85	This work

^1^ Two-dimensional electrochemical reactor. ^2^ Three-dimensional electrochemical reactor. ^3^ Heavy oil refinery. ^4^ Current density. ^5^ Reaction time. ^6^ NaCl concentration. ^7^ Granular activated carbon. ^8^ Electrode spacing.

**Table 5:** Factors and levels in the response surface experiment.

Factor	Variable	Low Level (g/L)	High Level (g/L)
Voltage	A	7	11
Time	B	1	3
Activated carbon loading	C	8	12

**Table 6:** Levels of each variable and corresponding COD_Cr_ removal efficiency obtained from the Box–Behnken design.

RUN	Coded Variable Level			Real Variable Level			COD_Cr_ Removal (%)	
	A	B	C	Voltage (V)	Time (h)	Activated Carbon Loading (g)	Predicted	Experimental
1	0	−1	−1	9	1	8	55.93	55.60
2	0	0	0	9	2	10	86.68	85.60
3	1	1	0	11	3	10	64.89	64.60
4	0	−1	1	9	1	12	56.65	56.90
5	0	0	0	9	2	10	85.68	85.70
6	−1	1	0	7	3	10	74.59	74.80
7	0	0	0	9	2	10	86.68	85.30
8	0	1	−1	9	3	8	69.45	69.20
9	−1	−1	0	7	1	10	58.11	58.40
10	0	0	0	9	2	10	85.68	86.00
11	1	−1	0	11	1	10	46.11	45.90
12	1	0	1	11	2	12	55.74	55.70
13	1	0	−1	11	2	8	49.86	50.40
14	0	1	1	9	3	12	78.38	78.70
15	0	0	0	9	2	10	85.68	85.80
16	−1	0	1	7	2	12	65.54	65.00
17	−1	0	−1	7	2	8	61.76	61.80

**Table 7:** ANOVA results for the COD_Cr_ removal.

Source	Sum of Squares	df	Mean Square	F-Value	*p*-Value
Model	3096.30	9	344.03	1671.80	<0.0001
A-Voltage	235.44	1	235.44	1144.13	<0.0001
B-Time	621.28	1	621.28	3019.07	<0.0001
C-Activated carbon time	46.56	1	46.56	226.26	<0.0001
AB	1.32	1	1.32	6.43	0.0389
AC	1.10	1	1.10	5.36	0.0538
BC	16.81	1	16.81	81.69	<0.0001
A^2^	1053.11	1	1053.11	5117.52	<0.0001
B^2^	336.52	1	336.52	1635.30	<0.0001
C^2^	570.48	1	570.48	2772.22	<0.0001

R^2^ = 0.9995, R^2^ (adjusted) = 0.9989; R^2^ (predicted) = 0.9938.

## Data Availability

The data presented in this study are available on request from the corresponding author.
